# Neuropsychological profile and clinical effects of arginine treatment in children with creatine transport deficiency

**DOI:** 10.1186/1750-1172-7-43

**Published:** 2012-06-19

**Authors:** Annamaria Chilosi, Manuela Casarano, Alessandro Comparini, Francesca Maria Battaglia, Margherita Maria Mancardi, Cristina Schiaffino, Michela Tosetti, Vincenzo Leuzzi, Roberta Battini, Giovanni Cioni

**Affiliations:** 1 Department of Developmental Neuroscience, IRCCS Stella Maris, Viale del Tirreno 331, 56128, Calambrone Pisa, Italy; 2 Division of Child Neuropsychiatry, University of Pisa, Pisa, Italy; 3 Unit of Child Neuropsychiatry, Giannina Gaslini Institute, Genoa, Italy; 4 Department of Pediatrics, Giannina Gaslini Institute, Genoa, Italy; 5 Department of Pediatrics and Child Neuropsychiatry, University of Roma “La Sapienza” Italy and Coordinator of GISMet-creatine Italian Group, Pisa, Genova, Roma, Italy

**Keywords:** Creatine transporter deficiency, XLMR, Speech delay, Arginine treatment, *SLC6A8* gene, Magnetic resonance spectroscopy

## Abstract

**Background:**

SLC6A8, an X-linked gene, encodes the creatine transporter (CRTR) and its mutations lead to cerebral creatine (Cr) deficiency which results in mental retardation, speech and language delay, autistic-like behaviour and epilepsy (CRTR-D, OMIM 300352). CRTR-D represents the most frequent Cr metabolism disorder but, differently from Cr synthesis defects, that are partially reversible by oral Cr supplementation, does not respond to Cr treatment even if precociously administrated. The precursors of Cr are the non-essential amino acids Glycine (Gly) and Arginine (Arg), which have their own transporters at the brain–blood barrier level and, therefore, their supplementation appears an attractive and feasible therapeutic option aimed at stimulating Cr endogenous synthesis and, in this way, at overcoming the block of Cr transport within the brain. However, until now the effects of Arg and/or Gly supplementation on Cr brain levels and behaviour have been controversial.

**Methods:**

In this study five Italian male patients affected by CRTR-D were supplemented with oral L-Arg at a dosage of 300 mg/kg/day divided into 3 doses, for 24–36 months. Biochemical and plasmatic amino acids examinations and thyroid hormone dosages were periodically performed. Moreover, Proton and Phosphorus Magnetic Resonance Spectroscopy (MRS) was monitored during follow-up in concurrence with neuropsychological evaluations.

**Results:**

During L-Arg treatment a clinical improvement in motor skills and to a lesser extent in communication and attention was observed. In addition, all patients had a reduction in the number and frequency of epileptic seizures. Daily living skills appeared also to be positively influenced by L-Arg treatment. Moreover, Total Cr and especially PhosphoCr, evaluated by proton and phosphorus spectroscopy, showed a mild increase, although well below the normal range.

**Conclusion:**

This study provides information to support the effectiveness of L-Arg supplement treatment in CTRT-D patients; in fact the syndromic pattern of cognitive and linguistic deficit presented by CRTR-D patients was partially altered by L-Arg supplementation especially at a qualitative clinical level. Oral L-Arg may represent not only a protective factor towards a further cognitive decline, but can lead to the acquisition of new skills.

## Introduction

Creatine Transporter Deficiency (CRTR-D) (MIM#300036) is a X-linked disorder caused by SLC6A8 gene mutation and represents the most frequent disorder of Creatine (Cr) metabolism [1-3] and one of the causes of X-linked mental retardation (XLMR) in a percentage varying from 2.1% to 3.5% in a European XLMR panel [4,5].

CRTR-D is characterized by mental retardation of variable degree, language deficit, behavioural disorders, epilepsy [6-13], cardiac manifestations [11] and gastrointestinal problems in adult patients [12]. Since first described in 2001 [6], more than 150 patients from 60 families and more than 20 different mutations have been identified [7,14-18] without determining a clear genotype-phenotype correlation.

Differently from Cr synthesis defects, where clinical symptoms may be partially reversed by oral Cr supplementation (AGAT deficiency, OMIM 602360) [19,20] or plus a regimen aimed at reducing Guanidino Acetate (GAA) accumulation (GAMT deficiency, OMIM 601240) [2,21,22], Cr supplementation resulted ineffective for CRTR-D [2,3,7,23,24], also when precociously provided [12]. Supplementation with L-arginine (L-Arg) alone [25,26] or in combination with Cr and/or L-glycine (L-Gly) has been described, although its efficacy is still controversial [27-29].

Few studies have described in detail the neuropsychological profile of patients affected by CRTR-D [8], in particular in terms of neuropsychological standardization tests [10,18,25,26,28,29]; the even fewer studies on adult patients, confirm the presence of mental retardation [11,30] or deterioration [12].

In this study we have investigated five Italian CTRT-D children in order to describe: a) their neuropsychological profile b) the effects of L-Arg supplementation on improving intellectual and behavioral disorders.

## Material and methods

### Patients

Five Italian male children with genetically confirmed CRTR-D diagnosed at IRCCS Stella Maris, Pisa (P1, P2) and IRCCS Gaslini, Genoa (P3, P4, P5) and regularly monitored at these two respective Children Hospitals were recruited for this study.

The diagnosis was suspected on the basis of an increased urine Cr/Creatinine ratio and further confirmed by the absence of Cr peak at proton magnetic resonance spectroscopy (^1^ H-MRS) and by *SLC6A8* gene examination. The pathogenetic role of the mutation was confirmed by the assessment of Cr uptake in fibroblasts or lymphoblasts. Clinical, laboratory and magnetic resonance findings had already been reported in previous publications on four patients [9,10,15,18].

The clinical and laboratory data are summarized in Table 1.

**Table 1:** Clinical and laboratory findings of the study sample

	**P1**		**P2**		**P3**		**P4**		**P5**	
	**8 yrs, 6 mo[Battini, 2007]**		**5 yrs, 5 mo[Battini, 2011]**		**21 mo[Schiaffino, 2005]**		**5 yrs[Mancardi, 2007]**		**17 yrs**	
**Clinical picture at diagnosis**	moderate mental retardation, severe language deficit withoral–motor dyspraxia, epilepsy, temper tantrums, clumsiness		mild mental retardation, language deficit		severe mental retardation, impairment in social interaction, epilepsy and language deficit		moderate mental retardation, epilepsy, language deficit		severe mental retardation, language deficit, epilepsy	
**Type of seizures**	sporadic occipital seizures		two episods of febrile convulsions at 3 and 4 yrs		myoclonic seizures (2 yrs) asymmetrical spasms at 8.5 yrs		severe epilepsy with SE and polymorphic seizures (generalized, focal, myoclonic); followed by sporadic GTCS		secondarygeneralized FS at onset; apparently primary GTCS from 8 to 16 yrs	
Urinary molar Cr to Crn ratio and Cr uptake*	Cr/Crn ratio: 2.35 (n.v. < 1.0) Cr uptake absent		Cr/Crn ratio: 3.08 (n.v. < 1.0) Cr up take absent		Cr/Crn ratio: 3.6 (n.v. 0.006-1.2) Cr uptake absent		Cr/Crn ratio:1.83 (n.v. 0.03-0.92) Cr uptake absent		Cr/Crn ratio: 2981 μmol Cr/mmol Crn(n.v. 22–1273) Cr uptake absent	
**SLC6A8 mutation**	c. 1006–1008 del AAC (de novo)		c.757 G > C (inherited)		IVS1-2A > G (inherited)		c.1631 C > T (inherited)		c. 1006–1008 del AAC (inherited)	
**MRI**	slight white matter hyperintensity in paratrigonal region		normal		white matter hyperintensity in the posterior regions		normal		white matter hyperintensity in the posterior regions	
**MRS**	**PCr/PDE**	**tCr**	**PCr/PDE**	**tCr**	**PCr/PDE**	**tCr**	**PCr/PDE**	**tCr**	**PCr/PDE**	**tCr**
	n.v.1.40 ± 0.07	n.v.4.37 ± 0.34	n.v.1.40 ± 0.07	n.v.4.37 ± 0.34	n.v.1.40 ± 0.07	n.v.4.37 ± 0.34	n.v.1.40 ± 0.07	n.v.4.37 ± 0.34	n.v.1.40 ± 0.07	n.v.4.37 ± 0.34
**T0**	0.16	0.65	0.78	2.01	0.86	0.3	-	-	-	-
**T1**	0.33	1.27	0.92	1.98	1.07	2.1	-	-	0.97	2.01
**T2**	0.58	1.88	#	#	#	#	0.98	2.97	0.95	1.7
**T3**	0.63	1.8					0.81	3.49	1.00	2.0
**Age at Arg treatment onset, dosage and duration of follow-up**	8 yrs, 6 mo 300 mg/kg bw/day per os24 mo		5 yrs, 5 mo300 mg/kg bw/day per os24 mo		8 yrs, 6 mo300 mg/kg bw/day per os24 mo		6 yrs, 5 mo300 mg/kg bw/day per os36mo		17 yrs300 mg/kg bw/day per os24 mo	

**Legend:***SE*: status epilepticus; *GTCS*: generalized tonic-clonic seizure; *FS*: febrile seizures; n.v.: normal values.

*Cr*: creatine. *Crn*: creatinine.

TO baseline time, at diagnosis; T1, T2, T3: after 12, 24 and 36 months of Arginine treatment.

*Cr uptake was performed in fibroblasts or lymphoblasts (see the reference indicated).

**#** not yet acquired.

The study was approved by the Ethical Committee of the IRCCS Stella Maris. Informed participation consent was obtained from all parents.

### Treatment and biochemical and spectroscopy monitoring

All patients were supplemented with oral precursor of Cr, L-Arg, at a dosage of 300 mg/kg/day divided into 3 doses, for 24 and 36 months. The rationale for the choice of L-Arg therapeutic trial was based on several considerations: i) Arg acts as substrate stimulating the synthesis of Cr at level of astroglial cells [31] and peripheral cells [32]; ii) every brain cell is able to uptake Arg [33,34], so ensuring its own local needs in Arg for Cr synthesis or other metabolic requests; iii) in comparison with Gly, clinical pharmacology of Arg is relatively well known in term of efficacy, dosages, routes of administration, and side effects in adults and children [35] and thus we ruled out the possibility to administer both precursors (Gly and Arg) in order to avoid possible confounding results. Finally we were discouraged from adding oral Cr supplementation considering the extremely low residual Cr transport we detected in the fibroblasts from our patient [10].

Blood and urine amino acids, ammonia, urea nitrogen, blood Cr and creatinine, alanine aminotransferase (ALT), aspartate aminotransferase (AST), creatine kinase (CK), lactate dehydrogenase (LDH), amylase, TSH, T3, T4, blood cell count and haemoglobin were periodically monitored in all patients. Moreover, brain Cr and Phosphocreatine (PCr) fluctuations under treatment were monitored respectively by means of Proton and Phosphorus MRS which were performed before (P1, P2, P3) and during Arg supplementation (P1, P2, P3, P4, P5), contemporarily with neuropsychological examinations. P4 and P5, followed to IRCCS Gaslini, were included in the study after two and one year of treatment respectively.

At Proton MRS total brain Cr (tCr) was measured in terms of absolute concentration, expressed in mmol/liter VOI (mM), using the LCModel. Phosphorus MRS was analyzed with a Spectra data processing software (SAGE/IDL) and PosphoCreatine (PCr) was measured in terms of ratio with respect to the phosphodiester signal (PDE) that was chosen as an internal reference since this metabolite is not included in any known metabolic circuit in cellular ATP production [25,36].

### Neuropsychological assessment

The children were submitted to a comprehensive neuropsychological evaluation, in particular: cognitive abilities were assessed by means of Griffiths Scales [37], an index of non-verbal intelligence derived from the combination of scores from the scales for Eye-Hand Coordination and Performance, while language evaluation included: productive vocabulary (the Italian version of the Mac Arthur-Bates Communication Development Inventories for Infants and Toddlers - CDI), Axia Vocabulary Test and receptive vocabulary (Peabody Picture Vocabulary Test, PPVT), sentence comprehension (Test of Comprehension of Grammar for children, TCGB), level of language organization in spontaneous speech and in sentence repetition test (see ref. 10 for a more detailed description and references of language assessment). Adaptive functioning in motor, communication daily living and socialization domains was also evaluated using the Vineland Adaptive Behaviour Scales (VABS).

Since not all patients were able to cooperate in the structured tests at baseline (T0), we reported their data from the first neuropsychological assessment during L-Arg treatment where cooperation was obtained. Neuropsychological evaluation was then repeated every year from T0, i.e. the beginning of treatment (T1 = 12 months, T2 = 24 months, T3 = 36 months). VABS data were instead available starting from T0 to follow-up study for all patients.

## Results

### Clinical data

During L-Arg treatment, we observed clinical improvement involving motor skills with acquisition of better dynamic coordination and only relative progress in communication and attention. Furthermore, there was a reduction in the number and frequency of epileptic seizures in all patients; in some cases (P4 and P5) it was possible to replace polytherapy with monotherapy, in P1 the antiepileptic drug administration was stopped and in P3 a mild electroclinical improvement was observed, although he had not been administered antiepileptic drugs for a long time by decision of the family.

No important side effects were observed in any patients during oral L-Arg supplementation with the exception of incidental episodes of diarrhea, mainly related to adaptation to L-Arg dosage. An increase of body weight during the treatment was noticed only in P1 while all patients showed an increase of muscular strength without clinical change of muscle mass.

During treatment total brain Cr (tCr) and, especially, phosphocreatine (PCr) showed a mild increase, more evident during the first months of Arg treatment and then remained quite stable throughout the other examinations, although well below the normal range (Table 1, line MRS) [25,36].

### Neuropsychological profile at basal time and during follow up

The Griffiths Scales revealed different degrees of intellectual disability varying from mild (P2) to moderate (P1, P4) and to severe (P5). In all patients the profiles were characterized by a significant discrepancy between non-verbal and verbal skills with lower scores in Hearing & Speech and Practical reasoning scales and better scores in Eye-Hand coordination and Performance Scales. Due to the severe cognitive impairment with autistic-like behaviour of patient P3 a systematic neuropsychological assessment was not possible; however, severe degree of cognitive impairment was apparent from spontaneous observation. A stability in all subscales was observed, at least during the three years of our follow up.

**Figure 1:**
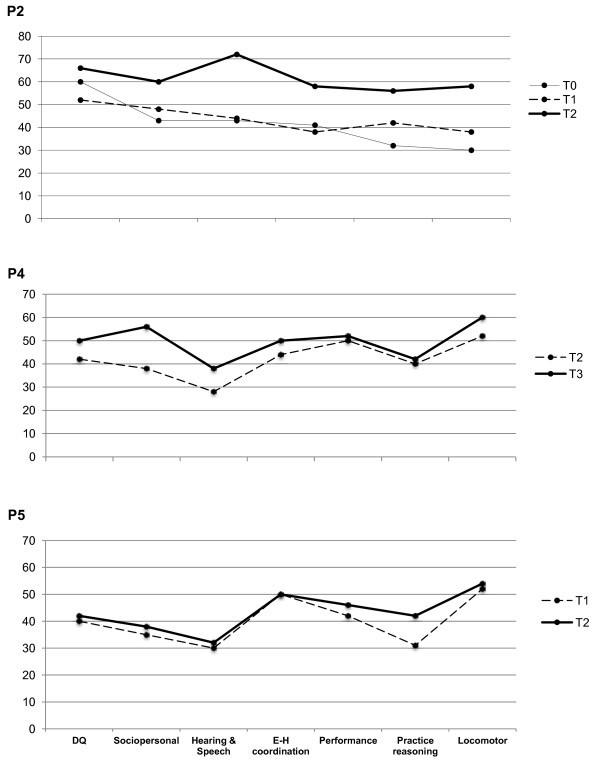
**Modifications in Griffiths Scale of three patients (P2, P4, P5) during treatment. **The results are expressed in equivalent age (EA). T0 = baseline; T1, T2, T3: after 12, 24, 36 months of L-Arg supplementation, respectively.

In Figure 1 we reported the modifications on Griffiths scale of three patients (P2, P4, P5) during treatment. P1 data had been already reported [25].

**Figure 2:**
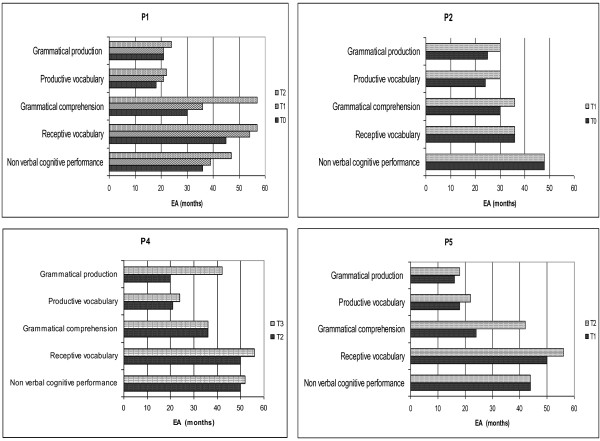
**Neuropsychological profiles of the four testable patients (P1, P2, P4, P5) during follow-up with oral arginine supplementation. **The results are expressed in equivalent age (EA). T0 = baseline; T1, T2, T3: after 12, 24, 36 months of L-Arg supplementation, respectively.

The neuropsychological profile of the patients (with the exception of P3) is reported in Figure 2. In order to compare the performance of different tests for each patient, the results were expressed in equivalent age (EA).

For all testable subjects the expressive language performance appeared markedly reduced in comparison to receptive lexicon and non-verbal abilities. Moreover within verbal domain, sentence comprehension, even if delayed, was more preserved with respect to expressive language measures.

For all children the most affected area was expressive language, even if this impairment was present in differing degrees: P1, P4, P5 demonstrated productive language limited to isolated words and simple combinatorial speech with some simple routine utterances integrated with some deictic, iconic and representative gestures. Signs of oral-motor and speech dyspraxia were also present, more prominent in P1 [10]. Language skills were more advanced in P2: spontaneous language was at the level of production of multiword utterances mostly morphologically incomplete for omission of free grammatical morphemes and errors of bound morphology [18]. In P3 language was almost absent with sporadic production of echolalic and non-communicative speech.

During L-Arg treatment the four testable patients experienced a transformation in language skills: in particular, it was interesting to note that after the first two years of treatment P1, P2 and P5 showed an increment in grammatical comprehension of 18 (P2, P5) and 27 EA months (P1), while P4’s grammatical production improved by more than 20 EA months. These data appeared relevant also in comparison to the slow developing pace of children up to the beginning of the treatment. Interestingly, L-Arg supplementation seemed more effective in the verbal domain, particularly compromised in these patients, than in the cognitive non-verbal domain.

Even if a regular neuropsychological assessment was not obtained from P3, clinical observation revealed an improvement in eye contact, motor hyperactivity, attention span, and repetitive behaviour.

**Figure 3:**
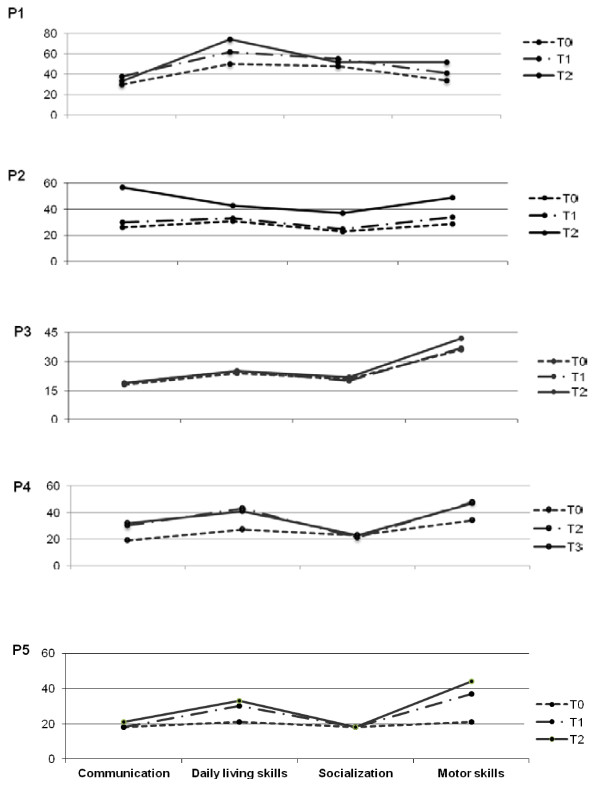
**VABS profiles in all five patients. **After L-Arg supplementation there was an improvement in adaptive skills, more significant for motor and daily living skills. The results are expressed in equivalent age (EA). T0 = baseline; T1, T2, T3: after 12, 24, 36 months of L-Arg supplementation, respectively.

As indicated in Figure 3, VABS showed, even if in various degrees of difficulty, a similar profile concerning adaptive skills in all patients. This profile was characterized by relatively better skills in daily living and motor abilities scales and lower abilities in communication and socialization, with the exception of P1 who showed a socialization skills score just under daily living skills. After L-Arg supplementation VABS profiles demonstrated an improvement in adaptive skills in all patients, more evident for motor skills, with the exception of P3. In particular, P2 and P4 showed an increment of 20 months and 13 EA months respectively, greater than expected in the lapsed period of one year, especially considering the previous pace of development. Daily living skills also appeared to be positively influenced by L-Arg: P1, P2 and P4 showed an increment of 24, 12 and 14 months respectively. On the contrary socialization skills seemed to receive no or little advantage from the L-Arg supplementation with the exception of P2 who showed a 14 month improvement; likewise communication skills also demonstrated reduced advantages, with the exception of P2 and P4 who showed a remarkable improvement of 31 and 11 months respectively.

## Discussion

The present study was designed to describe the pattern of neuropsychological derangement in CRTR-D patients and to examine stability or changes in intellectual functioning during the treatment with L-Arg. Several factors (*SLC6A8* mutation, age at diagnosis and at start of supplementation treatment, environment, co-morbidity and compliance) could contribute to the complexity of the CRTR-D clinical picture and in particular could influence the responsiveness to treatment.

Our CRTR-D patients’ profile appeared characterized by language deficits with dyspraxia and/or severe expressive language deficit with lexical receptive skills more advanced with respect to their mental age. There was a relative sparing of vocabulary comprehension in the context of different degree of mental retardation.

The first signs of the disease occurred in all patients in the first year of life with psychomotor and language delay, but the CRTR-D diagnosis always arrived later (mean age at diagnosis 7.6 yrs). Developmental course was characterized by a long phases of ‘development stagnation’ resulting in very restricted expressive language skills, cognitive and behavioural deficits, despite early speech and psychomotor therapy.

Most CRTR-D patients of previously published series [28,29] were described as affected by a pervasive developmental disorder (PDD) or PDD not otherwise specified (PDD-NOS) (i.e. they showed signs of autism spectrum). Conversely, only one of our cases (P3) showed PDD-NOS. The presence of PDD probably made neuropsychological evaluation by standardized tests difficult, as in our patient. The clinical differences and the difficulties in testing might partially account for the somehow better response to treatment reported in our studies.

Clinical variability between CRTR-D patients may be due to their genetic abnormalities; in our series, two patients (P1 and P5) showed the same mutation (Table 1) and presented a more severe language phenotype than the others. This same mutation was previously reported by Clark [17] in a patient with Attention Deficit and Hyperactivity Disorder (ADHD). One of our cases (P4) showed the same mutation identified in other two cases of Van de Kamp et al. [28] and in one case of Fons et al. [26]. In these patients the clinical picture was mainly characterized by mixed receptive-expressive language disorder variously associated to moderate mental retardation and epilepsy (P4), -PDD-NOS and ADHD [28], and mild mental retardation [26].

Epileptic seizures represent an important feature of CRTR-D patients relevant for the severity of their mental disability. L-Arg supplementation could have a positive role on seizure treatment as described in our patient (P3) and a more effective seizure control in association with antiepileptic drugs (P1, P4, P5) [27].

The decision to treat our children with oral L-Arg supplementation was based on several considerations, already discussed in the section of treatment and spectroscopy monitoring. L-Arg acts as substrate stimulating the synthesis of Cr at the astroglia cell level and, because every brain cell is able to uptake L-Arg [33], it is ensured its own local needs of L-Arg for Cr or other metabolic synthesis. Supplementation treatment with L-Arg alone for only nine months was chosen by Fons et al. [26] while in other studies a combination treatment with Cr, L-Arg and L-Gly with various dosage and timing was preferred [27-30]. The variable study protocols and the clinical heterogeneity of the various reported cohorts, especially for age at start of treatment, could explain the different treatment responsiveness and CRTR-D outcomes.

We found similar results for Proton and Phosphorus MRS in all our studied patients: Cr and PCr peak showed mild increase during Arg treatment (Table 1) and, although these data are different from other reported [28,29], worthy of attention and their significance should be addressed in further future studies.

Moreover, we observed not only a relatively stable pattern of neuropsychological functioning over time, but also an improvement in adaptive skills, mainly in daily living and in motor abilities. as possible cause of motor improvement. In addition, in some of our children, Developmental Quotient scores were available from the first year of life and before diagnosis; in these cases a decline in scores was observed, conversely during L-Arg supplementation a stability or slight improvement of mental age was found. An effect of repeated testing could be reasonably excluded, since the intervals between evaluations were sufficiently long.

Also the subject institutionalized from the age of 10 years (P5) who had a late diagnosis (17 yrs), a severe epilepsy and a greater decline of behavioural and intellectual skills, showed a mobilization of the functional profile after L-Arg supplementation, with improvement especially in receptive language and motor and daily living skills.

Only patient P3 with severe epilepsy, not treated for a long time by decision of the family, PDD-NOS and a delayed start of L-Arg treatment (about 7 yrs after diagnosis) did not show any quantitative behavioural changes after L-Arg supplementation.

The degree of behavioural change induced by L-Arg is probably related to other variables, such as the presence of varying degrees of drug-resistant epilepsy.

In Conclusion, this study provides information to support the validity of L-Arg supplementation treatment in CTRT-D patients; in fact the syndromic pattern of cognitive and linguistic deficit presented by CRTR-D patients can be partially changed by L-Arg supplementation especially at a qualitative clinical level. Oral L-Arg seems to represent not only a protective factor towards a further cognitive decline, but can possibly facilitate the acquisition of new skills.

The course of this disorder seems thus to be partially modifiable through the synergy of L-Arg treatment together with family compliance and administration of antiepileptic drugs, potentially making this syndrome a partially treatable disorder, especially when diagnosis is precocious.

## Abbreviations

CRTR-D: Creatine Transporter Deficiency; Cr: Creatine; XLMR: X-linked mental retardation; AGAT: Arginine-glycine amidino transferase; GAA: Guanidino acetate Acid; GAMT: Guanidino acetate methyl transferase; L-Arg: L-Arginine; L-Gly: L-Glycine; H-MRS: Proton Magnetic Resonance Spectroscopy; ALT: Alanine aminotransferase; AST: Aspartate aminotransferase; CK: Creatine kinase; LDH: Lactate dehydrogenase; MRS: Magnetic Resonance Spectroscopy; VABS: Vineland Adaptive Behaviour Scales; tCr: Total Creatine; PCr: Phosphocreatine; EA: Equivalent age; PDD: Pervasive developmental disorder; PDD-NOS: Pervasive developmental disorder not otherwise specified; ADHD: Attention Deficit and Hyperactivity Disorder; MR: Mental retardation.

## Competing interests

The Authors report no financial conflict of interest.

## Authors’ contributions

AMC participated to the study concept and design, analysed and interpretated the data, drafted the manuscript and partecipated to a critical revision of the manuscript for important intellectual content. 

MC acquired, analysed and interpretated the data and drafted the manuscript. 

AC acquired, analysed and interpretated the data and drafted the manuscript. 

FMB acquired, analysed and interpretated the data. 

MMM acquired, analysed and interpretated the data. 

CS acquired, analysed and interpretated the data. 

MT acquired, analysed and interpretated the data. 

VL partecipated to the study concept and design and drafted the manuscript; partecipated to a critical revision of the manuscript for important intellectual content and supervised the study. 

RB participated to the study concept and design and analysed the data; drafted the manuscript and collaborated to a critical revision of the manuscript for important intellectual content and supervised the study. 

GC participated to the study concept and design; collaborated to a critical revision of the manuscript for important intellectual content and supervised the study. 

All authors read and approved the final manuscript.
